# Weekly SARS-CoV-2 Sentinel Surveillance in Primary Schools, Kindergartens, and Nurseries, Germany, June‒November 2020

**DOI:** 10.3201/eid2708.204859

**Published:** 2021-08

**Authors:** Martin Hoch, Sebastian Vogel, Laura Kolberg, Elisabeth Dick, Volker Fingerle, Ute Eberle, Nikolaus Ackermann, Andreas Sing, Johannes Huebner, Anita Rack-Hoch, Tilmann Schober, Ulrich von Both

**Affiliations:** Bavarian Health and Food Safety Authority, Oberschleissheim, Germany (M. Hoch, S. Vogel, V. Fingerle, U. Eberle, N. Ackermann, A. Sing);; Ludwig-Maximilians-University, Munich, Germany (L. Kolberg, E. Dick, J. Huebner, A. Rack-Hoch, T. Schober, U. von Both);; German Center for Infection Research, Munich (U. von Both)

**Keywords:** severe acute respiratory syndrome coronavirus 2, SARS-CoV-2, coronaviruses, viruses, coronavirus disease, COVID-19, sentinel, surveillance, primary schools, kindergartens, nurseries, childcare, respiratory infections, zoonoses, Germany

## Abstract

We investigated severe acute respiratory syndrome coronavirus 2 infections in primary schools, kindergartens, and nurseries in Germany. Of 3,169 oropharyngeal swab specimens, only 2 were positive by real-time reverse transcription PCR. Asymptomatic children attending these institutions do not appear to be driving the pandemic when appropriate infection control measures are used.

Children have been disproportionately affected by public health measures in the current coronavirus disease (COVID-19) pandemic (*1*). In contrast to other age groups, children have shown lower rates of severe acute respiratory syndrome coronavirus 2 (SARS-CoV-2)‒positive cases; lower risk for symptomatic, acute, COVID-19; a generally milder course of disease with the exception of some rare manifestations and the post–COVID-19 multisystem inflammatory syndrome in children; and lower secondary attack rates (*2*–*4*). Susceptibility to infection in <10 years of age is estimated to be lower than that for teenagers. Accumulating evidence shows that, given limited infection control measures, SARS-CoV-2 might spread sustainably in secondary/high schools but to a lesser degree in primary schools and nurseries (*2*,*5*).

Closure of childcare facilities and schools has been shown to negatively affect the physical and emotional well-being of children, teenagers, and parents, potentially having a long-term impact on their lives (*6*). Thus, various expert groups called for avoiding closing of these institutions (*7*,*8*). Against the background of presymptomatic transmission found in adults, it is critical to public health authorities to be able to rely on real-life data monitoring the number of asymptomatic yet infected children attending educational institutions (*9*). Some studies have reported low numbers of infected cases in primary schools or childcare facilities but were conducted during a lockdown or semi-lockdown period (*5*,*10*). The aim of our study (the Münchner Virenwächter Study) was to implement a real-time sentinel program in a representative number of 5 primary schools and 5 (6 in phase 2) nurseries/kindergartens in Munich, Germany.

## The Study

**Figure 1:**
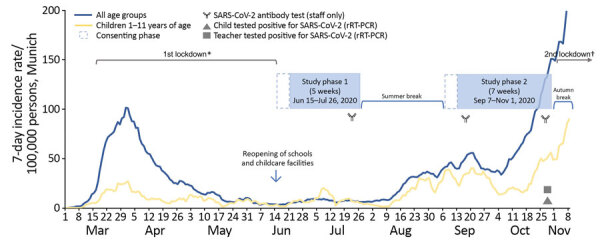
Weekly SARS-CoV-2 sentinel surveillance in primary schools, kindergartens, and nurseries, Germany, June‒November 2020.Timeline of Münchner Virenwächter study in context of pandemic activity in Munich, Germany. The 7-day incidence rates were derived from the national surveillance system according to the German Infection Protection Act, Bavarian Health and Food Safety Authority as of November 28th, 2020. rRT-PCR, real-time reverse transcription PCR; SARS-CoV-2, severe acute respiratory syndrome coronavirus 2. *Included closure of schools and childcare facilities. †Schools, childcare facilities, and shops/businesses kept open.

This study was approved by the ethics committee of the Ludwig-Maximilians University under project no. 20-484. We intended to accomplish a timely detection of infected cases and offer an additional level of safety to participating institutions during regular operating mode. The study spanned over 2 phases (Figure 1): phase 1, June 15‒July 26, 2020; and phase 2, September 7‒November 1, 2020. Participating institutions were randomly selected, and written informed consent was obtained in the first week of each phase. To correct for underrepresentation of younger children (<5 years of age), we included an additional nursery/kindergarten into phase 2.

We tested oropharyngeal swab specimens for SARS-CoV-2 by using real-time reverse transcription PCR (rRT-PCR); weekly samples were obtained from randomly selected children (n = 20) and staff (n = 5) in each institution. Swab specimens were taken on-site by trained medical personnel, and results were timely reported. For rRT-PCR, we processed specimens by using the AmpliCube Coronavirus SARS-CoV-2 Panel (Mikrogen, https://www.mikrogen.de) on a CFX96 Touch rRT-PCR Detection System (Bio-Rad, https://www.bio-rad.com). We retested single gene results by using the Xpert Xpress SARS-CoV-2 Test (Cepheid, https://www.cepheid.com).

We performed SARS-CoV-2 IgG screening at 3 sequential time points for samples from consenting staff members by using the Liaison SARS-CoV-2 S1/S2 IgG System (DiaSorin, https://www.diasorin.com) (Figure 1). We confirmed active results by using the RecomLine SARS-CoV-2 IgG Lineblot (Mikrogen). Antibody screening was complemented by obtaining a throat swab specimen at the same time to exclude active infection. Institutions were asked to respond to a questionnaire assessing implementation of infection control measures for phases 1 and 2.

**Figure 2:**
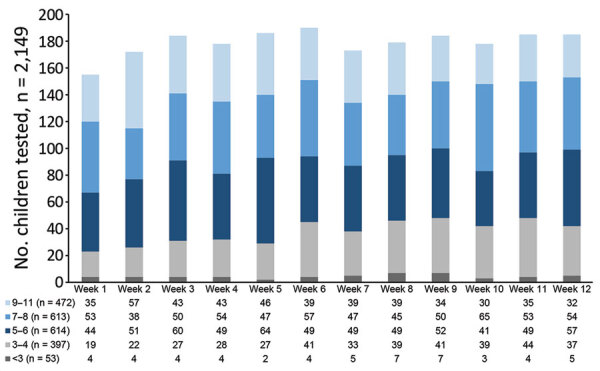
Weekly SARS-CoV-2 sentinel surveillance in primary schools, kindergartens, and nurseries, Germany, June‒November 2020. Distribution of weekly pediatric oropharyngeal swab samples for SARS-CoV-2 testing by real-time reverse transcription PCR. Color code indicates individual age groups. Age stratification is per week of children tested for SARS-CoV-2. SARS-CoV-2, severe acute respiratory syndrome coronavirus 2.

We processed 3,169 oropharyngeal swab specimens during the 12-week testing period, 2,149 from children (median age 7 years, range 1‒11 years, male:female ratio 1.03) and 1,020 from staff (median age 41 years, range 17–76 years, male:female ratio 0.13). We also obtained 493 swab specimens from staff during weekly testing and 527 swab specimens to complement serologic testing. We also tested 527 blood samples from staff for SARS-CoV-2 IgG. We determined pediatric sample distribution per study week (Figure 2).

No SARS-CoV-2 infections were detected during phase 1 of the study. During phase 2, only week 12 yielded 2 positive samples from 1 primary school. All SARS-CoV-2 IgG test results were negative at timepoints 1 and 2; only 1 positive serologic result was detected at timepoint 3. We identified some changes for implemented infection control measures between study phases and for individual facets between schools and childcare facilities (Table). All children attending primary schools were wearing face masks on school premises, except when seated for classes. Regular ventilation was begun as a daily routine in all institutions, as per national infection prevention and control guidance (Robert Koch Institute, https://www.rki.de).

**Table:** Comparison of implementation of infection control measures for phase 1 and 2 during weekly SARS-CoV-2 sentinel surveillance in primary schools, kindergartens, and nurseries, Germany, June‒November 2020*

Infection control measure	Childcare facilities		Primary school	
	Phase 1	Phase 2	Phase 1	Phase 2
Reduced number of supervised children	0/5	0/6	5/5	0/5
Supervision of children by rotating groups/classes	0/5	0/6	5/5	0/5
Physical distancing between staff members inside	5/5	6/6	5/5	5/5
Physical distancing between staff members outside	5/5	6/6	5/5	4/5
Physical distancing between children inside	1/5	2/6	5/5	5/5
Physical distancing between children outside	2/5	2/6	5/5	1/5
Face mask for staff members inside	0/5	6/6	4/5	5/5
Face mask for staff members outside	0/5	2/6	4/5	4/5
Face mask for staff members during drop-off/collection of children	3/5	6/6	4/5	5/5
Face mask for parents during drop-off/collection of children	5/5	6/6	4/5	5/5
Parents allowed to enter premises when dropping off or collecting children	5/5	4/6	1/5	2/5
Washing hands before collection of children by parents	3/5	5/6	1/5	1/5
Use of bathroom facilities separate for individual groups/classes	5/5	5/6	2/5	2/5
Closure of garden/playground areas	0/5	0/6	0/5	0/5
Use of garden/playground areas separate for individual groups/classes	4/5	4/6	5/5	5/5
Handwashing before meals	5/5	6/6	5/5	5/5
Handwashing before entering classes/groups	5/5	5/5	5/5	5/5
Hand disinfectant dispensers provided on premises	4/5	6/6	4/5	3/5
Cancellation of common activities	5/5	6/6	5/5	5/5

*Values indicate number of participating childcare facilities or primary schools that implemented a specific infection control measure, which ranged from 0/5 to 5/5 or 0/6 to 6/6. Phase 1, June 15‒July 26, 2020; phase 2, September 7‒November 1, 2020. SARS-CoV-2, severe acute respiratory syndrome coronavirus 2.

Designed during the first lockdown in Munich, our study was intended to determine a feasible SARS-CoV-2 sentinel program in primary schools and childcare facilities in anticipation of a second pandemic wave and increasing incidence rates. Although public health and political authorities were concerned that childcare institutions would be major drivers of the pandemic, our results suggest that this did not happen. This result was consistent with those of another report suggesting that it was unlikely that children are major drivers of the pandemic even if attending schools (*11*).

Our study was not powered to accurately illustrate changes in incidences during low-incidence periods because of small sample sizes. However, we detected 2 cases in a primary school, 1 child and 1 teacher, during a high, local, 7-day incidence rate of 50 cases/100,000 children 1–11 years of age and 150 cases/100,000 persons in the general population. Tracing of 36 close contacts (33 classmates and 3 private contacts) identified only 1 additional case in another asymptomatic child in the same class. Telephone interview‒based contact tracing showed that the teacher reported to have experienced unspecific symptoms of headache and malaise 6 days before testing. Thus, it seems reasonable to deduce that transmission occurred from staff to both children.

## Conclusions

Several reports have assessed the role of children in the dynamics of SARS-CoV-2 transmission. A study conducted in day care centers in Germany that used buccal mucosal and anal swabs for SARS-CoV-2 detection concluded that day care centers are not relevant reservoirs in a low prevalence setting (*12*). However, this study used self-testing, lacked oropharyngeal swab specimens, and was conducted during a minimal local incidence rate. Our study covered both low and high 7-day incidence periods while obtaining oropharyngeal swab specimens from children 1‒11 years of age. Ismail et al. reported complementary data from the United Kingdom, which showed that staff members had an increased risk for SARS-CoV-2 infection compared with students in any educational setting and that most cases linked to outbreaks were in staff (*13*).

Secondary attack rate analysis of the cases in our study also suggests that infections were transmitted from staff to children. In addition, low prevalence for SARS-CoV-2 antibodies in staff over the 3-month study period suggests no relevant infection activity in neither work nor private setting. Another recent report highlighted the need for maintaining low infection rates in the community to keep schools open during the pandemic (*14*).

Our study was conducted before the emergence of SARS-CoV-2 variants, such as B.1.1.7. Thus, the effect of this variant on children could not be addressed in our study. However, recent data from the United Kingdom found no evidence of more severe disease in children during the second wave, suggesting that infection with the B.1.1.7 variant does not result in a greatly different clinical course than the original strain (*15*).

We conclude that asymptomatic children attending primary schools, kindergartens, and nurseries are not greatly contributing to pandemic distribution of SARS-CoV-2 while adhering to infection control measures described above, even during high local background incidence. Thus, these children are unlikely to initiate clusters or outbreaks in the community when these institutions continue to play their critical role for the physical and emotional well-being of children and their families.
